# Optogenetic activation of septal GABAergic afferents entrains neuronal firing in the medial habenula

**DOI:** 10.1038/srep34800

**Published:** 2016-10-05

**Authors:** Kyuhyun Choi, Youngin Lee, Changwoo Lee, Seokheon Hong, Soonje Lee, Shin Jung Kang, Ki Soon Shin

**Affiliations:** 1 Department of Biology, Department of Life and Nanopharmaceutical Sciences, Kyung Hee University, Seoul, Republic of Korea; 2 Department of Molecular Biology, Sejong University, Seoul, Republic of Korea

## Abstract

The medial habenula (MHb) plays an important role in nicotine-related behaviors such as nicotine aversion and withdrawal. The MHb receives GABAergic input from the medial septum/diagonal band of Broca (MS/DB), yet the synaptic mechanism that regulates MHb activity is unclear. GABA (γ -aminobutyric acid) is a major inhibitory neurotransmitter activating both GABA_A_ receptors and GABA_B_ receptors. Depending on intracellular chloride concentration, however, GABA_A_ receptors also function in an excitatory manner. In the absence of various synaptic inputs, we found that MHb neurons displayed spontaneous tonic firing at a rate of about ~4.4 Hz. Optogenetic stimulation of MS/DB inputs to the MHb evoked GABA_A_ receptor-mediated synaptic currents, which produced stimulus-locked neuronal firing. Subsequent delayed yet lasting activation of GABA_B_ receptors attenuated the intrinsic tonic firing. Consequently, septal GABAergic input alone orchestrates both excitatory GABA_A_ and inhibitory GABA_B_ receptors, thereby entraining the firing of MHb neurons.

The MHb plays an important role in nicotine-related behaviors such as nicotine aversion and withdrawal^1^,^2^,^3^. Neurons in the ventral two-thirds of the MHb comprise cholinergic subdivision of the nucleus: These neurons densely express cholineacetyltranferase (ChAT) and nicotinic acetylcholine receptors^4^,^5^. Typically, they exhibit spontaneous tonic firings that are not synchronized with other adjacent neurons^4^. A few factors have been known to affect the tonic firings of MHb neurons. *Ex-vivo* application of nicotine increases firing frequency *via* neurokinin-2 signaling^6^. In addition, HCN channels appeared to be involved in the tonic firing^4^. More importantly, it was found that changes in MHb neuronal tonic firing is associated with the somatic and affective signs of nicotine withdrawal^4^. GABA, glutamate and ATP are known to be released from afferents to the MHb. Retrograde labeling revealed that MS/DB is a main GABAergic source and the triangular septum that comprises the posterior septal structure is glutamatergic and purinergic sources of the MHb^7^,^8^,^9^.

In the mammalian brain, GABA is known to inhibit neuronal activity *via* both GABA_A_ and GABA_B_ receptors. Ionotropic GABA_A_ receptors activation elicits inward Cl^−^ flows, which inhibit neuronal activity by generating hyperpolarizing inhibitory postsynaptic currents (IPSCs). GABA_B_ receptors, a member of inhibitory G-protein coupled receptors, stabilize neuronal activity through a couple of different pathways. For instance, GABA_B_ receptors activate Gα_io_ proteins, which in turn inhibit adenylyl cyclase *via* Gα_io_ or gate ion channels *via* G_βγ_^10^,^11^. A growing body of evidence suggests that depending on intracellular Cl^−^ concentration, neurons can be depolarized by GABA-generating inward currents through GABA_A_ receptors. In the dentate gyrus, a well-known neurogenic region, neural stem cells are depolarized by GABA_A_ receptors^12^,^13^,^14^,^15^. In the developing brain, GABA_A_ receptors are excitatory until 2 postnatal weeks^15^. Recurrent seizures induce excitatory GABA signal by downregulating K^+^/2Cl^−^ cotransporter (KCC2)^16^,^17^ and upregulating Na^+^/K^+^/2Cl^−^ cotransporter 1 (NKCC1)^18^. In an acute stress mouse model, noradrenergic receptor-mediated KCC2 downregulation removes GABA_A_ receptor-mediated synaptic inhibitory constraint of the parvocellular neuron in the hypothalamus^19^. Therefore, under the certain circumstances, GABA plays as an excitatory neurotransmitter.

Interestingly, it has been known that lack of KCC2 in MHb neurons results in a high internal chloride concentration^20^,^21^. Therefore, GABAergic transmission in the MHb may function in excitatory and inhibitory manners *via* GABA_A_ receptors and GABA_B_ receptors, respectively. Given that MHb neurons generate spontaneous tonic firing^4^,^20^, we asked whether this rhythmic firing can be modulated by MS/DB GABAergic inputs. In the present study, exploiting the optogenetic controls of MS/DB input to the MHb, we showed that GABA released from MS/DB afferents entrains MHb neuronal firing by orchestrating both excitatory GABA_A_ receptors and inhibitory GABA_B_ receptors.

## Results

### Spontaneous tonic firing in MHb neurons

**Figure 1:**
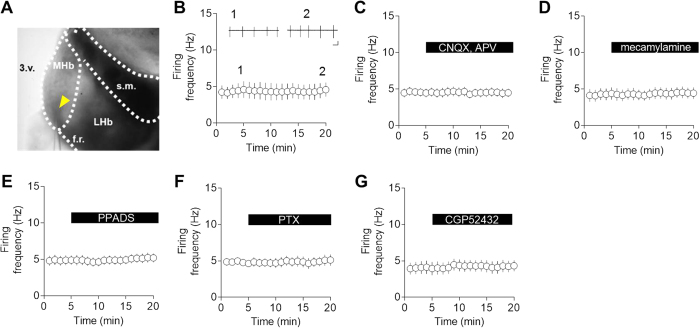
Tonic firing is intrinsically generated in the MHb. (**A**) Representative image showing recording site from acute brain slice visualized using infrared-DIC optics. The yellow arrowhead points to the tip of patch pipette. (**B**) Time course of tonic firing with loose-seal cell-attached recording in aCSF (n = 7). Inset shows representative traces of tonic firing recorded at time indicated by the numbers (Scale bars, 100 pA and 100 ms). (**C**) Tonic firing was not changed in the presence of CNQX (10 μM) and APV (30 μM). n = 6. (**D**) Tonic firing was not changed in the presence of mecamylamine (10 μM). n = 5). (**E**) Tonic firing was not changed in the presence of PPADS (50 μM). n = 6. (**F**) Tonic firing was not changed in the presence of picrotoxin (100 μM). n = 4. (**G**) Tonic firing was not changed in the presence of CGP52432 (10 μM). n = 5.

MHb neurons lack KCC2 expression^20^,^21^. Given that KCC2 is critical for extruding Cl^−^ in adult neurons^15^,^22^, intracellular Cl^−^ concentration in MHb neurons is likely to be higher. Therefore, to obtain stable intrinsic firing frequency without disturbing the intracellular ionic composition, loose-seal cell-attached recordings were made with aCSF-filled patch pipettes^23^. Recordings were performed at the ventral region of the MHb as shown in Fig. 1A and stable action currents with average firing frequency of 4.36 ± 1.06 Hz were consistently observed. When we measure any time-course change of the firing rates, the paired Student’s t-test showed no significant difference between the initial value (0–5 min) and late value (15–20 min) (aCSF: 99.99 ± 6.57% compared with baseline, n = 7; Fig. 1B), indicating that stable recordings can be maintained for at least 20 min. The MHb receives GABAergic, glutamatergic and purinergic inputs^7^,^8^,^9^. In addition, the MHb has marked expression of nicotinic receptors^2^,^5^. Thus, we first examined whether the synaptic inputs are involved in the tonic firing of MHb neurons. Inhibition of glutamatergic (AMPA receptor antagonist, CNQX 10 μM; NMDA receptor antagonist, D-APV 30 μM), cholinergic (nACh receptor antagonist, mecamylamine 10 μM), purinergic (P2X receptor antagonist, PPADS 50 μM) or GABAergic (GABA_A_ receptor antagonist, picrotoxin 100 μM; GABA_B_ receptor antagonist, CGP52432 10 μM) inputs did not modify the spontaneous tonic firing (Fig. 1). When the firing rates recorded 10–15 minutes after the drug treatments were compared with baseline firing rates (0–5 min), the paired Student’s t-test revealed no significant effect of treatments on percent changes in the firing rates (CNQX and D-APV: 98.88 ± 1.70, n = 6; mecamylamine: 107.60 ± 3.54, n = 5 ; PPADS: 107.6 ± 2.54, n = 6; picrotoxin: 100.70 ± 6.54%, n = 4; CGP52432: 107 ± 3.69, n = 5). The results demonstrate that spontaneous tonic firing is independent of these synaptic inputs.

**Figure 2:**
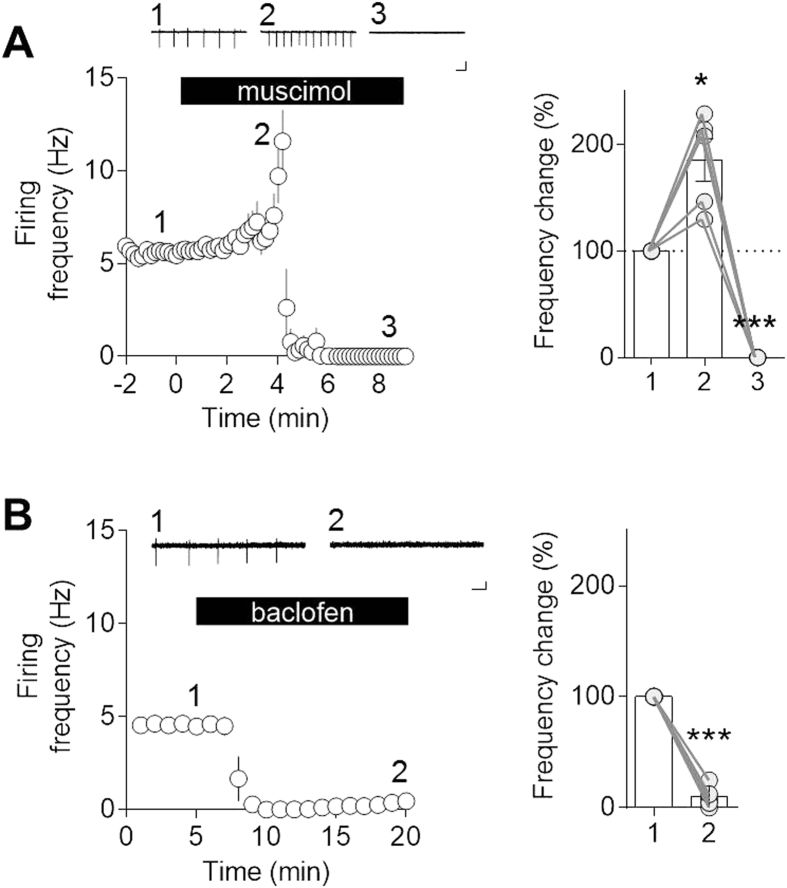
GABA_A_, and GABA_B_ receptors regulate MHb neuronal firing in opposite ways. (**A**) Muscimol (10 μM) briefly increased tonic firing and then completely abolished it. Left: Time-course change in firing frequency. Inset shows representative traces recoded at time indicated by the numbers. Scale bars represent 100 pA and 100 ms, respectively. Right: Percent change in firing frequency was compared with the baseline value obtained as in the left panel. Asterisks denote significant difference compared with the baseline value. *P < 0.05 and **P = 0.0013, repeated measure one-way ANOVA and Bonferroni’s post-hoc test, n = 5. (**B**) Baclofen (10 μM) completely abolished tonic firing. Left: Time-course change in firing frequency. Inset shows representative traces recoded at time indicated by the numbers. Scale bars, 100 pA and 100 ms. Right: Percent change in the firing frequency was compared with the baseline value. Asterisks denote significant difference compared with the baseline value. *P < 0.0001, paired Student’s t-test, n = 5.

Now, we speculated that activation of GABA_A_ receptors elicits excitatory postsynaptic currents (EPSCs) in MHb neurons due to the lack of KCC2 proteins thereby enhances MHb neuronal activity. As expected, muscimol (10 μM), a GABA_A_ receptor agonist, showed significant effect on firing frequency (F_1, 4_ = 65.75, P = 0.0013, one-way repeated measure ANOVA). As shown in Fig. 2A, tonic firing was briefly increased 185.3 ± 19.8% in the early stage of muscimol application (P < 0.05 compared with baseline firing frequency, Bonferroni’s post-hoc test) and quickly became quiescent (P < 0.001 compared with baseline firing frequency, Bonferroni’s post hoc test). This might be due to the sodium channel inactivation as a result of prolonged membrane depolarization caused by lasting excitatory GABA-mediated currents.

Meanwhile, GABA_B_ receptor is G-protein coupled receptor that associates with pertussis toxin sensitive Gi/o family, that in turn regulates specific ion channels and cAMP cascades^10^,^11^. Consequently, activation of GABA_B_ receptors stabilizes neuronal activity. As shown in Fig. 2B, baclofen (10 μM), a GABA_B_ receptor agonist, markedly blocked MHb neuronal firing (9.83 ± 4.32% compared with baseline firing rates, p < 0.0001, n = 5, paired t-test). The result is consistent with the well-known inhibitory effect of GABA_B_ receptors on neuronal excitability. Taken together, while GABA may not be involved in basal tonic firing of MHb neurons (Fig. 1), activation of GABA transmission would actively modify neuronal firing. Therefore, we next tested the possibility that GABAergic inputs to the MHb efficiently regulate neuronal tonic firing in the MHb.

### The MS/DB GABAergic input to the MHb

**Figure 3:**
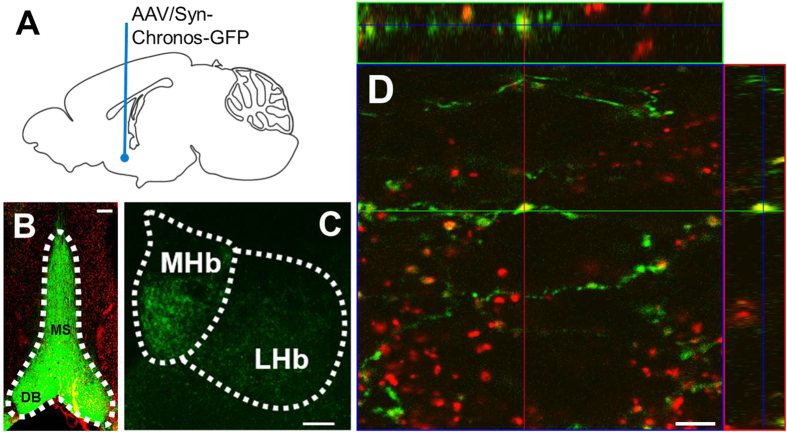
Direct projection of MS/DB GABAergic neurons to the MHb. (**A**) AAV Syn::Chronos-GFP delivered into the MS/DB. (**B**) Confocal image showed the robust expression of Chronos-GFP in the MS/DB, the AAV injection site. Scale bar: 200 μm. (**C**) Confocal image showed the expression of Chronos-GFP in the MHb, the MS/DB projection site. Scale bar: 100 μm. (**D**) Scanning confocal microscopy revealed that Chronos-GFP-expressing MS/DB afferents (green) were immunostained for vGAT1 (red) in the MHb. Scale bar: 5 μm.

Previous histological study using retrograde tracers show that GABAergic neurons in MS/DB project to the MHb^7^. However, functional GABAergic connections between these two regions have not been examined. Using optogenetic approaches, we tried to elucidate the functional properties of the synapses connecting between the MS/DB and the MHb. We delivered AAV expressing Chronos-GFP [Syn:: Chronos-GFP] into the MS/DB (Fig. 3A). Four to six weeks post infection, marked GFP signals were observed in the injection site (Fig. 3B). In addition, significant GFP signals were also apparent in the ventral MHb (Fig. 3C). As expected, the GFP-positive axon collaterals from the MS/DB showed the expression of vGAT1, a GABAergic presynaptic marker (Fig. 3D).

**Figure 4:**
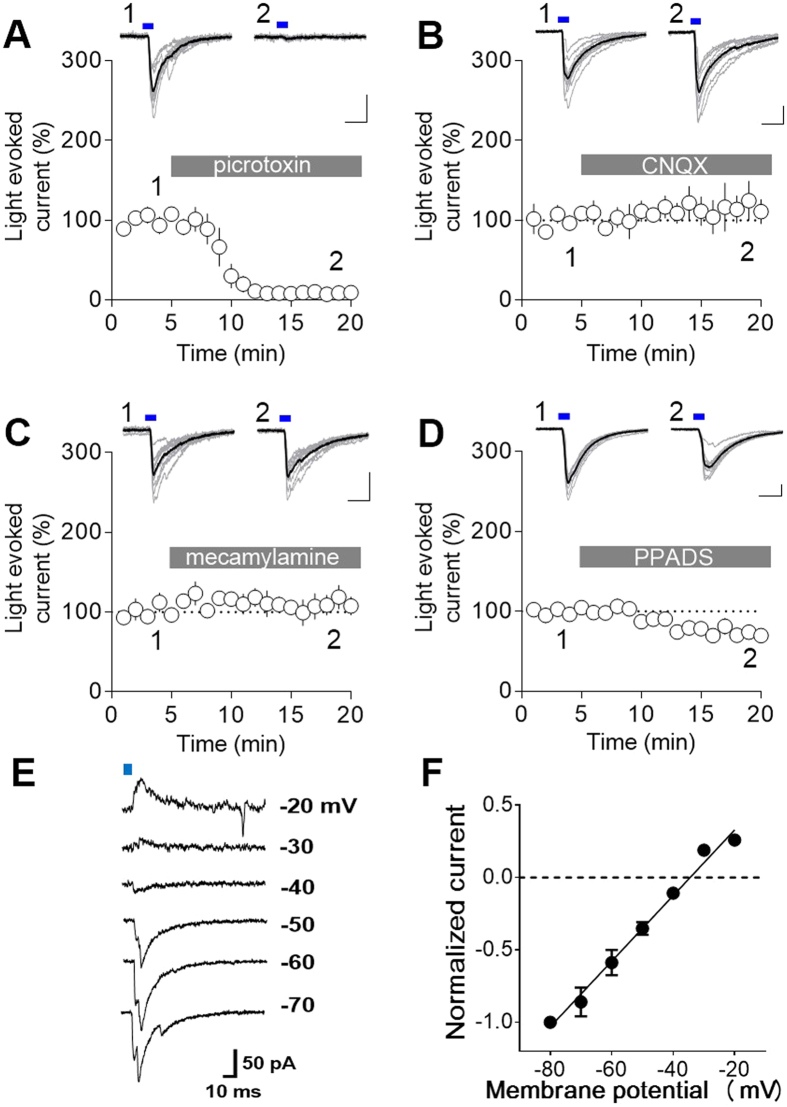
Optogenetic stimulation of Chronos-expressing MS/DB afferents evoked GABA_A_ receptor-mediated currents in the MHb. (**A–D**) Light stimulation (470 nm, 5 ms indicated by the blue bar) evoked postsynaptic current at −70 mV in whole-cell mode. Representative light-evoked current traces (top, scale bars: 50 pA and 10 ms) and time course changes of the light-evoked currents (bottom) upon treatments with various receptor antagonists were shown. (**A**) Picrotoxin, 100 μM, n = 4; (**B**) CNQX, 10 μM, n = 5; (**C**) mecamylamine, 10 μM, n = 5; (**D**) PPADS, 50 μM, n = 5; ***P < 0.0001 and **P = 0.0038, paired Student’s t-test. (**E,F**) Gramicidin perforated patch recordings revealed depolarized E_GABA_ of MHb neurons. Representative recordings of the light-evoked (470 nm, 10 ms indicated by the blue bar) currents at the indicated membrane potentials shown to the right of each trace (**E**). *I-V* relationship of normalized light-evoked currents and the linear regression fit to the data points, n = 4 (**F**).

We next examined functional synaptic connectivity of the MS/DB GABAergic input to the MHb. Whole-cell recordings were obtained from MHb neurons and synaptic neurotransmitter release, putatively GABA, was triggered by illuminating Chronos with a blue LED (470 nm, 5-ms duration). Light stimulation produced inward current at −70 mV (Fig. 4). As expected, picrotoxin (100 μM) dramatically abolished the light-evoked currents (8.97 ± 3.54% compared with baseline currents, P < 0.0001, paired Student’s t-test, n = 4). These data indicate that GABA released from MS/DB inputs induces GABA_A_ receptor-mediated currents in the MHb. However, the light-evoked currents were unchanged with AMPA receptor antagonist (CNQX 10 μM: 114.0 ± 21.39% compared with baseline currents, P = 0.55, paired Student’s t-test, n = 5). In addition, non-specific nAChR blocker did not modify the light-evoked currents (mecamylamine 10 μM: 108.2 ± 13.43% compared with baseline, P = 0.57, paired Student’s t-test, n = 5). Interestingly, P2X receptor antagonist, PPADS (50 μM), slightly but significantly decreased the currents (77.76 ± 3.34% compared with baseline, P = 0.0038, paired Student’s t-test, n = 5). At present, it remains to be examined how purinergic transmission exerts its effect on GABAergic synaptic transmission.

Next we measured the reversal potential of the GABA_A_ receptor-mediated currents (E_GABA_) elicited by light stimulation. To preserve intracellular Cl^−^ concentration, gramicidin perforated patch recordings were performed. Figure 4E shows representative current traces evoked by a brief light stimulation (470 nm, 10-ms duration) at different membrane potentials: The light-evoked Cl^−^ currents were reversed in polarity between −40 mV and −30 mV. We then constructed current-voltage (*I-V*) plots for the Cl^−^ currents and used linear regression analysis to measure E_GABA_ (Fig. 4F). E_GABA_ was estimated to be −33.27 ± 1.43 mV (n = 4), which was more depolarized than resting membrane potential of MHb neurons (−44.50 ± 1.29 mV, n = 12). Therefore, activation of GABA_A_ receptors is expected to produce EPSCs in MHb neurons.

Although it has been reported that GABA_A_ receptor signaling is absent in the MHb^21^, our data clearly verify the functional GABA_A_ receptor-mediated synapses in the MHb. As we assessed the expression of mRNA encoding GABA_A_ receptor subtypes in the MHb, multiple subtypes appeared to be expressed in the MHb (Supplementary information Fig. S1).

### Entrainment of MHb neuronal firing *via* both GABA_A_ and GABA_B_ receptors

**Figure 5:**
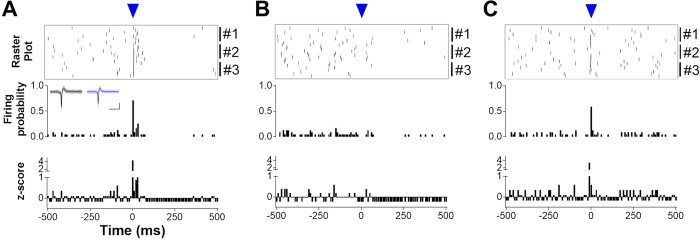
Optogenetic stimulation of Chronos-expressing MS/DB afferents evoked stimulus-locked firing in MHb neurons. A single light-stimulus (470 nm, 5 ms indicated by the blue arrowhead) evoked neuronal firing in loose-seal cell-attached mode. (**A**) Raster plot (top, 10-ms bin), corresponding normalized firing probability (middle) and z-score (bottom) profiles showed light-evoked firing. After the stimulation, spontaneous tonic firing was subsequently suppressed (24 epochs from 3 cells; the numbers on the right indicate three different cells). Inset demonstrates representative firing traces from spontaneous tonic (black trace) and light-evoked (blue trace) firings (Pearson correlation coefficient r = 0.95, P < 0.0001). Scale bars: 20 pA and 5 ms. (**B**) In the presence of picrotoxin (100 μM), a light stimulus failed to induce firing, with delayed quiet period still maintained (24 epochs from 3 cells). (**C**) In the presence of CGP52432 (50 μM), light stimulus induced neuronal firing, with spontaneous tonic firing not suppressed (24 epochs from 3 cells).

Our immunohistochemical and optogenetic approaches demonstrate that the MS/DB exerts GABAergic transmission on MHb neurons. Since agonists for GABA_A_ receptors increased MHb neuronal firing (Fig. 2A) and E_GABA_ was more depolarized than resting membrane potential of MHb neurons, we tested whether the MS/DB GABAergic inputs can generate firing in MHb neurons. To this end, we delivered light stimulus (470 nm, 5 ms) to activate Chronos expressed in MS/DB afferents upon loose-patch cell-attached recordings. Raster plot, corresponding normalized firing frequency and z-score profiles demonstrated that a single light stimulus reliably induced neuronal firing with the kinetics not different from those of the spontaneous tonic firing (Fig. 5A inset, Pearson correlation coefficient r = 0.95, P < 0.0001). A light stimulus frequently triggered a brief burst (Fig. 5A), which might be attributed to the light-evoked strong depolarization. Given the high input resistance of MHb neurons (1.29 ± 0.11 GΩ, n = 12), we expected that EPSPs generated by light-activated GABA_A_ currents are sufficient to trigger action potentials in MHb neurons. Indeed, the light-induced firing was completely abolished by picrotoxin (Fig. 5B). Meanwhile, a light stimulus not only induced immediate firing, but also generated delayed but prolonged quiet periods when spontaneous tonic firing was suppressed (as judged by negative values of z-score, Fig. 5A). Intriguingly, the suppression of tonic firing was still maintained in the presence of pricrotoxin. This might be due to the delayed but lasting activation of GABA_B_ receptors. Indeed, CGP52432 rendered the quiet period less prominent without affecting light-induced firing (Fig. 5C). Consequently, GABA_A_ and GABA_B_ receptors cooperatively participate in resetting the intrinsic tonic firing of MHb neurons.

**Figure 6:**
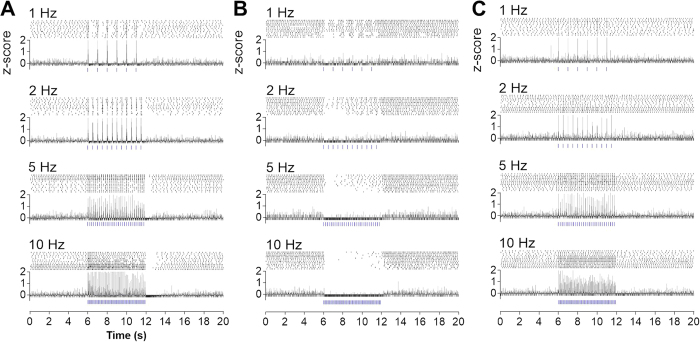
GABAergic MS/DB inputs entrained MHb neuronal firing. Light-stimulation (470 nm, 5 ms indicated by the blue bars) with various frequencies (1 Hz, 2 Hz, 5 Hz and 10 Hz for 3 sec) evoked neuronal firing in loose-seal cell-attached mode. (**A**) Light-stimulation generated the firing entrained to the stimulation frequency. Top: Representative raster plot from 3 cells as indicated by the numbers on the right (10-ms bin); bottom: Corresponding z-score profile (1 Hz, n = 40 epochs from 10 cells; 2 Hz, n = 40 epochs from 10 cells; 5 Hz, n = 28 epochs from 7 cells; 10 Hz, n = 36 epochs from 9 cells). (**B**) In the presence of picrotoxin (100 μM), light stimulation did not produce firing entrainment (1 Hz, n = 28 epochs from 7 cells; 2 Hz, n = 28 epochs from 7 cells; 5 Hz, n = 20 epochs from 5 cells; 10 Hz, n = 28 epochs from 7 cells). (**C**) In the presence of CGP52432 (50 μM), light stimulus induced entrainment without elimination of spontaneous tonic firing (1 Hz, n = epochs from 8 cells; 2 Hz, n = 28 epochs form 7 cells; 5 Hz, n = 28 epochs from 7 cells; 10 Hz, n = 32 epochs from 8 cells).

We next sought to establish whether various frequency activation of GABAergic input from the MS/DB can affect firing output in MHb neurons. When light with various frequencies (1–10 Hz) were applied, raster plot and corresponding z-score profile showed that MHb neurons reliably followed the stimulation frequency in a stimulus-locked manner, regardless of their original tonic firing frequency (Fig. 6A). To ensure the involvement of both GABA_A_ and GABA_B_ receptors on the firing entrainment, we examined the effects of picrotoxin and CGP52432. As expected, treatment of picrotoxin completely failed light-evoked firing, consequently abolished the firing entrainment (Fig. 6B). Instead, prominent light-induced suppression of intrinsic tonic firing was apparent in the presence of picrotoxin, which might be due to the activation of GABA_B_ receptors alone. In contrast, light stimulations in the presence of CGP52432 induced limited entrainment: light elicited GABA_A_ receptor-dependent firing, with less suppressed intrinsic tonic firings between stimulations (Fig. 6C). For this reason, CGP52432 rendered low-frequency entrainment (1 and 2 Hz) far less accurate. In conclusion, GABA_A_ and GABA_B_ receptors activated by GABA released from MS/DB afferents entrain MHb neuronal firing by exerting opposite effects on neuronal activity.

## Discussion

In the present study, we have demonstrated functional GABAergic synaptic connectivity between the MS/DB and the MHb. More importantly, we revealed that the GABAergic transmission alone is sufficient to entrain rhythmic firing in the MHb. We hope that our findings will give insight to understand MHb activity-mediated behaviors.

It has been known that MHb neurons possess spontaneous tonic firing^4^,^20^. A recent study revealed that MHb neurons are equipped with hyperpolarization-activated cyclic nucleotide-gated (HCN) channels that confer them with intrinsic rhythmic firing^4^. Indeed, the tonic firing is maintained without synaptic input as shown in this study (Fig. 1) as well as the previous observations^4^,^20^. This intrinsically generated tonic firing was dramatically modified following the activation of GABA_A_ and GABA_B_ receptors using the agonists, muscimol and baclofen, respectively: GABA_A_ receptor activation by muscimol triggered robust firings in MHb neurons, whereas GABA_B_ receptor activation by baclofen completely abolished the firings (Fig. 2). Previous study also reported GABA_A_ excitation in the MHb from juvenile rats (18- to 25-day old)^20^. Since GABA_A_ receptors are excitatory until 2 postnatal weeks in the developing brain^6^, the possibility was raised that the GABA_A_ excitation in the MHb only reflects the immature property of the developing brain. However, we consistently observed GABA_A_ excitation in the MHb obtained from adult mice (10–16 weeks), indicating that excitatory GABAergic activity is not attributed to immaturity of MHb neurons.

Both input and output pathways of the MHb have been well established^6^,^7^,^8^,^24^,^25^. Nevertheless, many functional studies related with the MHb have been focused on the output pathway^26^,^27^,^28^ or the habenular nucleus itself^1^,^2^,^4^,^29^,^30^. Indeed, only one study, to our knowledge, has been attempted to explain functional relevance of posterial septal afferents to the MHb^31^. Here we focused on MS/DB afferents to the MHb to establish functional input pathway of the MHb. It has been reported that the MHb received GABAergic projection from MS/DB using a retrograde tracer^7^. Consistently, we observed that Chronos-GFP-expressing MS/DB afferents express vGAT1, a GABAergic presynaptic marker. Furthermore, light stimulation of Chronos-GFP-expressing MS/DB afferents evoked picrotoxin-sensitive GABA_A_ currents, indicating functional synaptic connectivity of the GABAergic MS/DB afferents to the MHb. On the contrary, a previous study demonstrates that β 2/3 subtypes of GABA_A_ receptors are not expressed in the MHb and GABA application evoked no measurable currents in the MHb^21^. Our study, however, identified GABA_A_ receptor subtypes differentially expressed in the MHb using RT-PCR (Supplementary Fig. S1). More importantly, optogenetic stimulation evoked picrotoxin-sensitive GABA_A_ currents in MHb neurons (Fig. 4).

Meanwhile, it has been reported that GABA_B_ receptors are expressed substantially in MHb^20^ (Allen institute, experiment number 68862120, 71247614). Consistent with these observations, we found that baclofen completely abolished spontaneous tonic firing (Fig. 2B). Considering that GABA_A_ and GABA_B_ receptors exert the opposite effects on the activity of MHb neurons, we supposed that GABAergic MS/DB input alone entrains thereby synchronize MHb neuronal firings. We found that GABA optogenetically released from MS/DB afferents immediately elicited firing *via* fast activation of GABA_A_ receptors and subsequently suppressed intrinsic tonic firings *via* delayed but lasting activation of GABA_B_ receptors (Fig. 5). As a result, GABAergic MS/DB input entrained firing of MHb neurons (Fig. 6).

The MS/DB plays a key role in generating theta oscillations in the hippocampus^32^. And most MS/DB GABAergic neuronal firing is phase locked to hippocampal theta *in vivo*^33^. Intriguingly, hippocampal input to the MS/DB preferentially generates rhythmic firing of GABAergic neurons in the MS/DB^34^. In brain slices *ex vivo*, spontaneous tonic firings of MHb neurons are not synchronized with other adjacent neurons^4^. Taking into account the facts that MS/DB GABAergic neuronal firing is tuned to theta frequency *in vivo*^33^ and that GABAergic MS/DB input entrains neuronal firing in the MHb (Fig. 6), MS/DB input may synchronize MHb neuronal firing locked to theta rhythm *in vivo*. MHb neurons mainly project to the interpeduncular nucleus (IPN)^25^,^35^,^36^. Supposedly, unsynchronized intrinsic tonic firing in MHb neurons *per se* may produce subthreshold postsynaptic activity in IPN neurons. Now the synchronized MHb neuronal firing by MS/DB input may cause postsynaptic spatial summation, allowing IPN neurons to generate faithful suprathreshold activity.

Several studies have revealed that the MHb-IPN pathway play roles in nicotine-related behaviors. Activation of IPN GABAergic neurons that receive direct projection from the MHb triggers physical nicotine withdrawal symptoms^26^. Mice lacking nAChR α5 subunit exhibit decreased MHb input to IPN, which results in attenuated nicotine aversion^1^. Conversely, elevated expression of the nAChR β4 subunit increases nicotine aversion in mice by enhancing activity of the MHb to the IPN^2^. Experiments in animal models have demonstrated directly that the MHb-IPN pathway participates in nicotine withdrawal^37^. Therefore, it is plausible that MS/DB-MHb pathway also plays a role in nicotine-related behaviors because synchronized MHb neuronal firing would reliably activate IPN neurons. Although behavioral relevance still remains to be investigated, to our best knowledge, we have first demonstrated the MS/DB GABAergic entrainment of MHb neuronal firing.

## Material and Method

### Animals

Animal maintenance and treatment were carried out in accordance with the Animal Care and Use Guidelines issued by Kyung Hee University, Korea. All experiments with mice were performed according to the protocols approved by the Institutional Animal Care and Use Committee of Kyung Hee University (Approved protocol No. KHU(SE)-13-031, KHU(SE)-15-026). Male C57BL/6 mice (6–16 weeks of age, Orient Bio) were used for all experiments. Mice were group-housed on a 12-h:12-h light:dark cycle (light on 07:00) and had free access to food and water. The animals were held in a chamber with 20–24 °C, 30–60% humidity.

### Surgery

All streotaxic injections were performed under xylazine (5 mg/kg), tiletamine (60 mg/kg) and zolazepam (60 mg/kg) anesthesia using streotaxic frame (Stoelting). For virus injections, viral stocks (0.5 μL, AAV/Syn::Chronos-GFP, UNC vector core) were injected in the MS (AP +0.75, ML −0.05, DV −4.30) and DB (AP +0.75 mm, ML +−0.45 mm, DV −5.30 mm) using Picospritzer III (Parker) at a slow rate (50 nL/min). Mice were then allowed to recover for 4–6 weeks until further experiments.

### Acute Slice preparation

Mice were deeply anesthetized and performed cardiac perfusion with ice-cold aCSF of the following composition (in mM): 124 NaCl, 2.5 KCl, 1.2 NaH_2_PO_4_, 24 NaHCO_3_, 5 HEPES, 13 Glucose, 2 MgSO_4_, 2 CaCl_2_. After perfusion, the brain was quickly removed, submerged and coronally sectioned on a vibratome (VT1000s, Leica) to 250–300 μm in ice-cold aCSF. Slices transferred quickly to NMDG based recovery solution at 32 °C of the following composition (in mM): 92 NMDG, 2.5 KCl, 1.2 NaH_2_PO_4_, 30 NaHCO_3_, 20 HEPES, 25 glucose, 5 sodium ascorbate, 2 thiourea, 3 sodium pyruvate, 10 MgSO_4_, 0.5 CaCl_2_. After 10–12 min recovery periods, slices were transferred to room temperature aCSF chamber (20–22 °C) and left at least for 1 hour for the further recovery.

### Electrophysiology

Electrophysiological recordings were made using an EPC10 amplifier (HEKA elektronik). Patch-clamp pipettes were pulled (PP-83; Narishige) from borosilicate glass (Warner Instruments) and had a tip resistance of 3–6 MΩ when filled with internal solution. After recovery periods, acute slices were then transferred to the recording chamber, were fully submerged at a flow rate of 1.4–1.6 mL/min and maintained at 30 ± 1 °C in aCSF. Cells were visualized using epifluorescence and infrared differential interference contrast (IR-DIC) video microscopy with a 40X magnification water-immersion objective (BX51WI, Olympus). Tonic firings were measured in a loose cell-attached mode (8–25 MΩ) to prevent internal dialysis and aCSF was used for pipet solution. Chronos was stimulated by brief 470 nm light (5-ms duration) through the optic fiber (NA = 0.35) using light-emitting diode (LED; Doric lens, LEDC2) powered by an LED driver (Thorlabs, LEDD1B) under control of pulse generator (AMPI, Master-8). After the recordings, some slices were fixed in 4% paraformaldehyde in PBS to perform immunohistochemistry. Synaptic currents evoked by light were recorded at −70 mV in a whole-cell mode using pipet solution containing (in mM): 100 K-Gluconate, 20 KCl, 10 HEPES, 0.2 EGTA, 10 Na_2_-phosphocreatine, 4 MgATP, 0.3 Na_4_GTP; pH was adjusted to 7.2–7.3 with KOH. Pipet solution for gramicidin perforated patch recording contained (in mM) 140 KCl, 10 NaCl, 10 HEPES; pH was adjusted to 7.2–7.3 with KOH. Gramicidin (Sigma-Aldrich) was first dissolved in DMSO (10 mg/ml) to prepare a stock solution and then diluted to a final concentration of 10 μg/ml in the pipet solution. The gramicidin-containing solution was prepared and sonicated immediately before use. After E_GABA_ measurements, the integrity of the perforated patch was confirmed by rupturing the underlying sealed membrane and observing an abrupt change in access resistance and a shift in E_GABA_. Data were sampled at 10 kHz and filtered at 2.9 kHz with Bessel filter of the amplifier. Data were analyzed using Patchmaster (HEKA), Igor 6.0 (Wavemetrincs) or Minianalysis (synaptosoft).

### Immunohistochemistry

30 μm cryosected brain slices were permeabilized in 0.6% Triton X-100 and blocked in 3% normal donkey serum in PBS for 30 minutes in free floating condition. Rabbit anti-vGat1 antibody (1:500, Synaptic systems) was incubated for overnight in 1% normal donkey serum and 0.1% triton X-100 in PBS at 4 °C. For visualization, slices were incubated with Cy3-conjugated anti-rabbit secondary antibody (Jackson ImmunoResearch Laboratories) for 2 hours. Immunostained slices were scanned using a confocal laser microscope (LSM510, Carl Zeiss).

### RT-PCR

Total RNA was extracted using RNeasy Mini Kit (QIAGEN #74104) according to the manufacturer’s instruction. To synthesize first strand cDNA, 1 μg of total RNA was incubated at 70 °C for 5 min with 0.5 μg of oligodT and deionized water (up to 15 μl). The reverse transcription reaction was performed using 200 units of M-MLV reverse transcriptase (Promega, Madison, WI, USA) in 5X reaction buffer (250 mmol/l Tris-HCl; pH 8.3, 375 mM KCl, 15 mM MgCl_2_, 50 mM DTT), 28 units of RNasin inhibitor, and 2.5 mM dNTP mixtures at 42 °C for 90 min. The expression of GABA_A_ receptor subunits was examined by PCR as previously described^38^,^39^. Two microliters of the cDNA product were amplified in a mixture containing 5 pmole of GABAA receptor subtype-specific primers, 0.2 mM dNTPs and 1 unit *Taq* DNA polymerase (Promega, Madison, WI, USA) with reaction buffer in a final volume of 25 μl. The PCR amplification was carried out for 35 cycles of 94 °C for 30 sec, 52 °C for 30 sec and 72 °C for 1 min. The primers used were: beta-actin sense primer, 5′-TCACCCACACTGTGCCCATCTACGAG-3′; beta-actin anti-sense primer, 5′-GTGGTGAAGCTGTAGCCACGCTC-3′; GABA_A_ receptor subtype α1, α2, α3, α4, α5, α6, β1, β2, β3, γ1, γ2, γ3, δ primers were manufactured as previously described^38^. GABA_A_ receptor subtype ε, θ, ρ1, ρ2, ρ3 primer sequences were referred to in the previous paper^39^.

### Statistics

All data are presented as mean ± SEM. Data were analyzed by one-way analysis of variance (ANOVA) or paired Student’s t-test. Mean differences between groups were considered significant when P < 0.05. To quantify firing entrainment, loose seal cell-attached recording was normalized using firing probability and standard z-score transformation (bin size, 10 ms). Firing probability was calculated by following equations; sum of firing in each bins/total number of sweeps. Neuronal firing was normalized to the firing rates during 500 ms (Fig. 5) or 6 s (Fig. 6) just prior to light stimulus train.

## Additional Information

**How to cite this article**: Choi, K. *et al*. Optogenetic activation of septal GABAergic afferents entrains neuronal firing in the medial habenula. *Sci. Rep.* **6**, 34800; doi: 10.1038/srep34800 (2016).

## Supplementary Material

Supplementary Information
