# Supplementary material for: Optogenetic activation of septal GABAergic afferents entrains neuronal firing in the medial habenula
Source: Sci Rep. 2016 Oct 5;6:34800. doi: 10.1038/srep34800 (PMC5050514; doi:10.1038/srep34800)
Supplement: Supplementary Information [file srep34800-s1.pdf]

# Supplementary Information

**Optogenetic activation of septal GABAergic afferents entrains neuronal firing in the medial habenula**

Kyuhyun Choi,[1] Youngin Lee,[1] Changwoo Lee,[1] Seokheon Hong,[2] Soonje Lee,[1] Shin Jung Kang,[2] Ki Soon Shin[1,*]

[1]Department of Biology, Department of Life and Nanopharmaceutical Sciences, Kyung Hee University, Seoul, Republic of Korea; [2]Department of Molecular Biology, Sejong University, Seoul, Republic of Korea

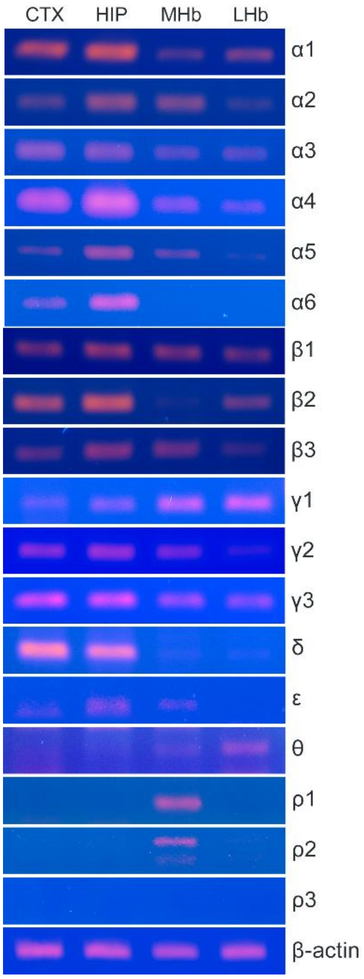


**Supplementary Figure S1. mRNA expression of $GABA_A$ receptor subtypes in different brain regions.** A representative example of the RT-PCR products specific for each GABAA receptor subtype mRNA is shown. CTX, cortex; HIP, hippocampus; MHb, medial habenula; LHb, lateral habenula. Detailed method is described in Materials and Method in the main text.
